# “From inflammation to fibrosis: a proposed role of mucosal-associated invariant T cells in lichen sclerosus – a conceptual framework”

**DOI:** 10.3389/fimmu.2026.1886594

**Published:** 2026-07-22

**Authors:** Marta Kasprowicz-Furmańczyk, Agnieszka Owczarczyk-Saczonek

**Affiliations:** School of Medicine, Collegium Medicum, University of Warmia and Mazury, Olsztyn, Poland

**Keywords:** fibrosing disease, inflammatory disease, lichen sclerosus, MAIT cells, mucosal-associated invariant T cells

## Abstract

Lichen sclerosus (LS) is a chronic inflammatory and fibrosing dermatosis with a well-documented autoimmune background and a dominant Th1/Th17-driven immune response. Despite growing insight into its immunopathogenesis, the mechanisms linking persistent inflammation to progressive fibrosis remain unclear. Mucosal-associated invariant T cells (MAIT cells) are innate-like lymphocytes enriched in epithelial tissues, characterized by rapid activation and robust production of proinflammatory cytokines. Although alterations in MAIT cells frequency and function have been described in autoimmune and fibrotic disorders, their role in LS has not yet been elucidated. This review integrates current knowledge on MAIT cells biology and proposes a conceptual framework for their potential involvement in LS, largely based on extrapolation from studies in other inflammatory and fibrotic conditions. We hypothesize that MAIT cells may contribute to disease progression by amplifying Th1/Th17 responses, promoting epithelial damage and indirectly supporting fibroblast activation through cytokine-mediated pathways, including the TGF-β axis. Furthermore, their responsiveness to microbial-derived ligands and inflammatory cytokines suggests a role in sustaining chronic immune activation within a dysregulated tissue microenvironment. Under chronic inflammatory conditions, MAIT cells may shift toward a proinflammatory and profibrotic phenotype, thereby contributing to the transition from inflammation to fibrosis. This hypothesis-generating framework requires experimental validation but may provide new insights into LS pathogenesis and potential therapeutic targets.

## 1 Introduction

Lichen sclerosus (LS) is a chronic, relapsing inflammatory skin disorder that most commonly involves the anogenital region, although extragenital lesions may also occur (1). It predominantly affects women, particularly in prepubertal and postmenopausal periods, but can also be observed in men. The estimated prevalence of LS varies depending on the population studied and diagnostic criteria, ranging from approximately 0.1% to 3%, and is likely underestimated due to underdiagnosis and delayed recognition (2). Clinically, LS presents with porcelain-white, atrophic plaques accompanied by pruritus, pain, and skin fragility, often leading over time to scarring, architectural changes, and functional impairment, including sexual and urinary dysfunction. The disease course is typically long-lasting and progressive, with a risk of fibrotic complications and, in some cases, malignant transformation to squamous cell carcinoma (3). Beyond physical symptoms, LS has a substantial negative impact on quality of life, contributing to psychological distress and reduced well-being (4). Current management relies primarily on high-potency topical corticosteroids, which are effective in controlling inflammation but do not fully prevent disease recurrence or the development of fibrosis in all patients (5). In this context, understanding how chronic inflammation evolves into irreversible tissue remodeling is clinically highly relevant, as it may open new avenues for more targeted and disease-modifying therapeutic approaches. Clinical and immunological data indicate a significant autoimmune component, as LS frequently coexists with other autoinflammatory diseases and is associated with autoantibody production (6). Current evidence supports a dominant Th1/Th17 immune response, leading to chronic inflammation and tissue remodeling (1, 3). However, the mechanisms linking the initial immune activation to progressive fibrosis remain unclear. Mucosal-associated invariant T cells (MAIT cells) are innate-like lymphocytes that respond rapidly to microbial metabolites and inflammatory cytokines (7). MAIT cells are abundant in mucous membranes and skin and are capable of rapidly producing IFN-γ, TNF-α, and IL-17 (8). Abnormalities in the number and function of MAIT cells have been described in autoimmune and fibrotic diseases, including systemic sclerosis (9) and oral lichen planus (10). Despite immunological similarities between LS and other autoimmune skin diseases, the role of MAIT cells in the pathogenesis of LS has not been analyzed. Due to their ability to produce proinflammatory mediators and modulate immune responses, they may represent a missing link between inflammation and fibrosis. This review summarizes current knowledge on MAIT cells biology and proposes a hypothesis regarding their potential involvement in the pathogenesis of LS. To our knowledge, this is the first conceptual framework linking MAIT cells to fibrosis in LS.

## 2 Immunopathogenesis of LS

LS is characterized by a dominant Th1-type immune response, with high levels of IFN-γ driving macrophage activation and cytotoxic CD8+ T-cell–mediated epithelial damage. In parallel, activation of the IL-17 axis contributes to keratinocyte stimulation, neutrophil recruitment, and amplification of local inflammation. Together, these pathways create a proinflammatory microenvironment that promotes chronic tissue injury and subsequent fibrotic remodeling (3). Skin lesions exhibit an intense inflammatory infiltrate, increased expression of adhesion molecules (ICAM-1) and chemokine receptors (including CXCR3, CCR5), and elevated levels of proinflammatory cytokines such as TNF-α, IL-1, IL-7, IL-15, and TGF-β (11). A humoral component, the presence of autoantibodies, is also demonstrated, further supporting the hypothesis of a loss of immune tolerance. Approximately 20–40% of LS patients suffer from other autoimmune diseases, such as autoimmune thyroiditis, vitiligo, or alopecia areata (12, 13). A positive family history, a female predominance, and a higher concordance in monozygotic twins indicate a significant genetic predisposition (13). Of particular importance are specific HLA alleles (e.g., HLA-DQ7, DQ8, DQ9, DR12), which may promote the presentation of autoantigens to autoreactive T cells and the perpetuation of chronic inflammation (14). Additionally, miR-155 overexpression is found in LS lesions, which enhances T cell activation and the production of proinflammatory cytokines while inhibiting immunoregulatory mechanisms (15). Accumulating evidence also indicates the involvement of the IL-17 axis. This cytokine, acting synergistically with TNF-α, activates keratinocytes, promotes neutrophil recruitment, and induces the expression of antimicrobial peptides such as S100A7, creating a self-perpetuating inflammatory loop (16). Chronic inflammation ultimately leads to tissue remodeling: loss of elastin fibers, disorganization of the extracellular matrix, and dermal fibrosis, processes mediated by TGF-β, GDF-15, increased MMP-2 and MMP-9 activity, and decreased ECM1 expression (1). As a result, LS represents a disease model in which a strong Th1/Th17 immune response progresses to persistent fibrosis, creating a microenvironment conducive to the activation of cells with pro-inflammatory and pro-fibrotic potential, such as MAIT cells.

## 3 MAIT cells biology

Mucosal-associated invariant T cells (MAIT cells) are an evolutionarily conserved population of T lymphocytes with innate-like features, combining properties of innate and adaptive immunity (8, 17). Through the TCRVα7.2 receptor, they recognize small metabolites derived from the riboflavin (vitamin B2) biosynthetic pathway of bacteria and fungi, presented by the major histocompatibility complex class I protein (MR-1) (10). They are most abundant in the blood and liver, but they also inhabit epithelial barriers in contact with the external environment – including the skin, oral cavity, intestines, respiratory tract and urogenital system (17). Their location just beneath the epithelium and their rapid ability to produce cytokines and cytotoxic mediators predispose them to the role of “guardians” of the integrity of tissue barriers (7).

### 3.1 Phenotypic characteristics

MAIT cells were originally defined based on the co-expression of the semi-variable TCRα chain Vα7.2–Jα33 (less frequently Jα20 or Jα12) and the lectin receptor CD161 in humans, in combination with a limited repertoire of TCRβ chains (including Vβ2, Vβ13, Vβ22) (18). In peripheral blood, most Vα7.2^+^CD161^+^ cells react with agonist liganded MR1 tetramers, confirming their classic MAIT cells phenotype (19).

The composition of the TCR receptor of blood MAIT cells is relatively homogeneous and in humans almost exclusively comprises the Jα33 segment. In contrast, mucosal tissues exhibit a greater diversity of Jα segments (e.g., Jα12, Jα27 in the intestine) and a greater variability of β-chains, suggesting a broader repertoire of specificities. These differences correlate with distinct transcriptomic and functional profiles between blood and tissue MAIT cells, indicating their specialized adaptation to the local immune environment (7).

In terms of co-receptor expression, CD8+ cells predominate in peripheral blood (~70%), with a smaller proportion of double-negative cells (DN, ~15%) and a small fraction of CD4+ cells (~5%) (20). In mucosal tissues, however, there is a relative enrichment of the DN population (40–50%, and in some individuals up to 80%) (21), with a reduced proportion of CD8+ cells. The female reproductive tract is an exception, where the distribution of the MAIT cells subpopulation is similar to that observed in blood, with a slight increase in the percentage of CD4+ cells in the endometrium (7, 22).

These data indicate that MAIT cells in blood and tissues differ in both TCR and coreceptor expression and functional profile, reflecting their distinct roles in immune surveillance in the circulation and at epithelial barriers. MAIT cells exhibit an effector memory phenotype (CD45RO^+^, CD27^+^, CCR7^-^, CD62Llow) and express multiple integrin receptors and homing cytokines (CCR5, CCR6, CXCR6, CCR9), which promote migration into inflammatory tissues (7).

In barrier tissues, MAIT cells often exhibit tissue-resident features (CD69^+^, CD103^+^). In healthy skin, up to 80–85% of MAIT cells can express cutaneous lymphocyte antigen (CLA), indicating their specialized ability to colonize the skin (23, 24). It is currently unknown whether tissue - resident MAIT cells can leave the mucosal tissues and recirculate (7).

### 3.2 Activation mechanisms

MAIT cells recognize metabolites derived from bacterial and fungal riboflavin biosynthesis (e.g., 5-OP-RU) as well as products of folic acid photodegradation, which are presented by the non-polymorphic MR1 molecule. Under inflammatory conditions, MR1 expression is upregulated both on professional antigen – presenting cells (APCs), including dendritic cells, monocytes, macrophages, and on epithelial cells (25).

Activation of MAIT cells follows a dual-signal model and may proceed via TCR-dependent or TCR-independent pathways (7). Classical activation involves TCR-mediated recognition of riboflavin-derived ligands presented by MR1 (25). This pathway is associated with the induction of tissue repair functions, as well as the production of IL-17 and IL-22, leading to moderate effector response. However, TCR-dependent signaling alone is often insufficient to elicit a fully developed cytotoxic response (25).

In the context of inflammation, MAIT cells can also be activated independently of TCR engagement through proinflammatory cytokines, particularly IL-12 and IL-18, as well as IL-7, IL-15, IL-23, and type I interferons (26, 27). The integration of TCR-dependent and cytokine-mediated signals results in robust effector activation, characterized by high-level production of Th1 (e.g., IFN-γ, TNF-α, IL-2), Th2 (e.g., IL-4, IL-10), and Th17 (e.g., IL-17, IL-22) cytokines, alongside increased expression of cytotoxic mediators such as granzymes (GzB, GzM) and perforin. This culminates in the acquisition of a strongly proinflammatory phenotype (28, 29). Under homeostatic conditions, MAIT cells activity is likely subject to tonic regulation by the microbiota and immune checkpoint pathways (including PD-1, TIGIT, LAG-3, and CTLA-4), thereby preventing excessive or uncontrolled activation (30).

### 3.3 Effector profile

Activated MAIT cells display a broad spectrum of effector functions, which are shaped by both the tissue microenvironment and the nature of the activating stimuli. They constitute an important early source of IFN-γ in inflamed tissues, where this cytokine promotes macrophage activation, enhances Th1 polarization, and inhibits the replication of intracellular pathogens; however, its sustained production may also contribute to tissue damage under chronic inflammatory conditions (7, 31). TNF-α produced by MAIT cells further amplifies the inflammatory response by supporting immune cell recruitment and pathogen control, although its excessive release may exacerbate epithelial injury and promote fibrotic processes (32). At epithelial barrier sites, MAIT cells frequently exhibit a functional bias toward IL-17 and IL-22 production. IL-17 contributes to the maintenance of barrier integrity through the regulation of tight junctions and facilitates neutrophil recruitment, yet its overproduction is associated with the persistence of chronic inflammation (24). Notably, the balance between IL-17 and IFN-γ production is highly context-dependent and varies according to tissue type (e.g., intestine, lung, skin) and the surrounding cytokine environment (33). In the resting state, MAIT cells exhibit limited cytotoxic activity, however, upon full activation, they upregulate the expression of cytotoxic mediators, including granzymes (GzB, GzM), perforin, and granulysin, and undergo degranulation, as indicated by increased CD107a expression. In this state, MAIT cells are capable of eliminating infected epithelial cells and macrophages but may also induce bystander damage to uninfected cells in an inflammatory environment, particularly under conditions of increased MR1 expression (7, 29).

## 4 MAIT cells in skin and mucosal tissues: from homeostasis to inflammation and fibrosis

In the skin, MAIT cells are mostly localized within the subepithelial compartment and dermis, where they display a tissue-resident phenotype (CD69^+^, CD103^+^, CLA^+^) and function as rapid sensors of microbial and tissue perturbations (21). Under homeostatic conditions, they support innate and adaptive immunity, maintain epithelial barrier integrity, and promote tissue repair programs, including wound healing and angiogenesis (34). During inflammation, MAIT cells redistribute from the circulation to affected tissues, where they accumulate and exhibit increased expression of activation markers such as CD69, HLA-DR, and CD38. Their recruitment is mediated by chemokine pathways, including the CCR6–CCL20 axis, and further reinforced by the local production of proinflammatory cytokines (21).

The functional outcome of MAIT cells activation reflects a dynamic balance between tissue repair and inflammation. Controlled, MR1-dependent activation promotes IL-17 and IL-22 production, supporting epithelial regeneration and restoration of tissue homeostasis (8). In contrast, a cytokine milieu enriched in IL-12, IL-18, type I interferons, and TNF-α drives strong MR1-independent activation, resulting in high IFN-γ and TNF-α production, enhanced cytotoxicity, and amplification of Th1-driven responses. This proinflammatory state contributes to sustained immune cell recruitment, chronic epithelial damage, and persistent lymphocytic infiltration (35). Prolonged activation may further lead to a state of functional exhaustion, determined by the expression of inhibitory receptors such as PD-1 and TIGIT; nevertheless, MAIT cells can remain a source of proinflammatory cytokines even in this state (36).

Collectively, MAIT cells represent a specialized T lymphocyte subset with pronounced functional plasticity and a dual role in antimicrobial defense and immunopathology. While they contribute to tissue homeostasis and repair under physiological conditions, their dysregulated activation in inflammatory environments may promote chronic inflammation and fibrosis. Accordingly, MAIT cells constitute a key component of the inflammation–repair–fibrosis axis, particularly in the context of inflammatory and fibrotic skin diseases (37).

### 4.1 MAIT cells in autoimmune and fibrosing diseases

Studies have demonstrated alterations in the number and function of MAIT cells in multiple autoimmune and inflammatory diseases, including systemic lupus erythematosus (SLE), systemic sclerosis (SSc), psoriatic arthritis, hidradenitis suppurativa, and lichen planus (10, 36). To date, no studies have been published evaluating MAIT cells in patients with LS (**Table 1**).

**Table 1:** MAIT cells in inflammatory diseases of the skin and connective tissue – implications for LS.

Disease	Circulating MAIT cells	Tissue infiltration	Functional profile	Implications for LS
Systemic sclerosis (9, 38–40)	Reduction (even 3-fold decrease in frequencies of Va7.2+CD161+ MAIT cells) Activated profile (CD69+MAIT cells) Exhaustive profile	–	IFN-γ, TNF- α	Potential involvement of MAIT cells in the fibrosis process
Oral lichen planus (10, 19, 41, 42)	Reduction Adults with recent-onset OLP: harbored, activated phenotype (increased frequency of CD69+ and CD38+ MAIT cells, elevated production of IL-17A), Adults with long-term OLP: activated and exhausted phenotype (high expression of CD69 and PD-1), increased production of the granzyme B	Increased frequencies of CD69 ^+^ MAIT cells and elevated secretion of pro‐inflammatory cytokines and cytotoxic molecules	IFN-γ, IL-17	Common Th1 inflammatory mechanism
Systemic lupus erythematosus (40, 43, 56)	Reduction Activated profile (CD69+MAIT cells) Exhaustion profile (PD1+, CD39+ and CTLA4+ MAIT cells)	Increased amount in mucosal and inflamed tissues Detection of MAIT cells in kidneys (lupus nephritis with class III and IV disease)	Proinflammatory	Migration of MAIT cells to inflamed tissue
Psoriatic arthritis (40, 57)	Reduction	Increased in synovial fluid (CD8+ MAIT cells) Proliferation on IL-23 stimulation Up-regulation of IL-23R	IL-17, IL-23	The role of MAIT cells in the IL-17/IL-23 axis
Lichen sclerosus	Unknown	Unknown	Unknown	A clear research gap

In diseases with a prominent fibrotic component, such as systemic sclerosis, significant changes in MAIT cell number and function have been reported, which may suggest their potential role in the inflammation-fibrosis axis (9, 38). These cells may influence fibrogenesis indirectly through the secretion of cytokines such as IFN-γ and TNF-α, which modulate the activity of fibroblasts and endothelial cells. In addition, persistent inflammation may promote secondary production of TGF-β by macrophages and epithelial cells – a key mediator of fibrogenic processes (44). Notably, the effects of IFN-γ and TNF-α on fibrosis are context-dependent: IFN-γ may exert antifibrotic effects by inhibiting fibroblast proliferation, yet under chronic inflammation it can still contribute to tissue remodeling.

Chronic MAIT cells activation may also promote epithelial damage, sustain local inflammation and influence macrophage polarization toward profibrotic phenotypes, enhancing TGF-β production and extracellular matrix deposition (45). Additionally, MAIT cells may contribute to a Th17-type environment through IL-17 production, supporting matrix remodeling and persistent fibrotic signaling via the IL-17–TGF-β axis (37). Their cytotoxic activity may further drive repeated cycles of epithelial injury and repair, favoring fibrosis through excessive fibroblast activation. Thus, MAIT cells likely contribute to a self-perpetuating loop of inflammation and dysregulated repair rather than acting as direct profibrotic effectors.

Overall, MAIT cells appear to play a context-dependent and predominantly indirect role in fibrotic processes, mediated through cytokine production, epithelial injury, and interactions with immune and stromal cells. These observations provide a framework for considering their involvement in diseases characterized by chronic inflammation and fibrosis, although their precise role likely depends on tissue context and disease stage.

## 5 Discussion: MAIT cells in LS – hypothesis

At present, there is no direct evidence demonstrating the involvement of MAIT cells in LS. The mechanisms discussed below should therefore be interpreted as a hypothesis, based on extrapolation from known MAIT cell biology and observations derived from other autoimmune, inflammatory, and fibrotic conditions.

In the pathogenesis of LS, integration of immune-mediated inflammation with processes leading to chronic fibrosis is increasingly recognized as essential. While multiple immune cell populations are capable of producing key cytokines implicated in LS (e.g., IFN-γ, TNF-α, IL-17, IL-22), MAIT cells warrant particular attention due to their distinct biological features. These include their enrichment at epithelial and mucosal sites, semi-invariant TCR-dependent recognition of microbial riboflavin metabolites presented by MR1, rapid responsiveness to inflammatory cytokines (IL-12, IL-18, IL-23) in a TCR-independent manner, and their tissue-resident, innate-like effector profile (7, 35). Taken together, these properties position MAIT cells at the interface between epithelial integrity, microbial sensing, and immune activation, making them particularly relevant in LS, which involves barrier disruption, chronic inflammation, and potential dysbiosis. In this context, MAIT cells may represent a functional link between these processes.

One key mechanism may be the amplification of the Th1 response. Upon activation, MAIT cells produce significant amounts of IFN-γ, a cytokine central to the Th1 axis. IFN-γ enhances the classic activation of macrophages (M1 phenotype), which begin to secrete TNF-α, IL-1β, and reactive oxygen species (46). This may contribute to tissue damage within the basal layer of the epidermis and to deepening of the inflammatory infiltrate. A positive feedback loop may develop in which tissue damage increases exposure of autoantigens, promoting further recruitment and activation of immune cells. Due to their rapid effector function and pre-activated phenotype, MAIT cells may amplify these responses earlier and more dynamically than conventional Th1 cells, thereby sustaining the dominant Th1 profile in LS rather than initiating it.

In parallel, MAIT cells may participate in the IL-17 axis. In an environment rich in IL-12, IL-18, or IL-23, they are capable of producing IL-17, which activates keratinocytes and fibroblasts to secrete chemokines (e.g., CXCL1, CXCL8), enhancing neutrophil recruitment (47). IL-17 also promotes further production of proinflammatory cytokines, maintaining a local inflammatory microenvironment and affecting epithelial barrier integrity under chronic conditions (48). Given their localization within epithelial tissues and responsiveness to local stress signals, MAIT cells may efficiently couple barrier disruption with sustained IL-17-driven inflammation and functionally complement Th17 cells.

Importantly, MAIT cells exhibit functional plasticity and have been implicated in tissue repair processes. Through the production of IL-17 and IL-22, they may support epithelial regeneration, barrier integrity, and antimicrobial defense. In early or acute settings, this function may be protective. However, under conditions of chronic stimulation, persistent activation may shift their role toward sustained inflammation, cytotoxicity, and tissue damage. This dual functionality highlights the context-dependent nature of MAIT-cell responses.

**Figure 1:**
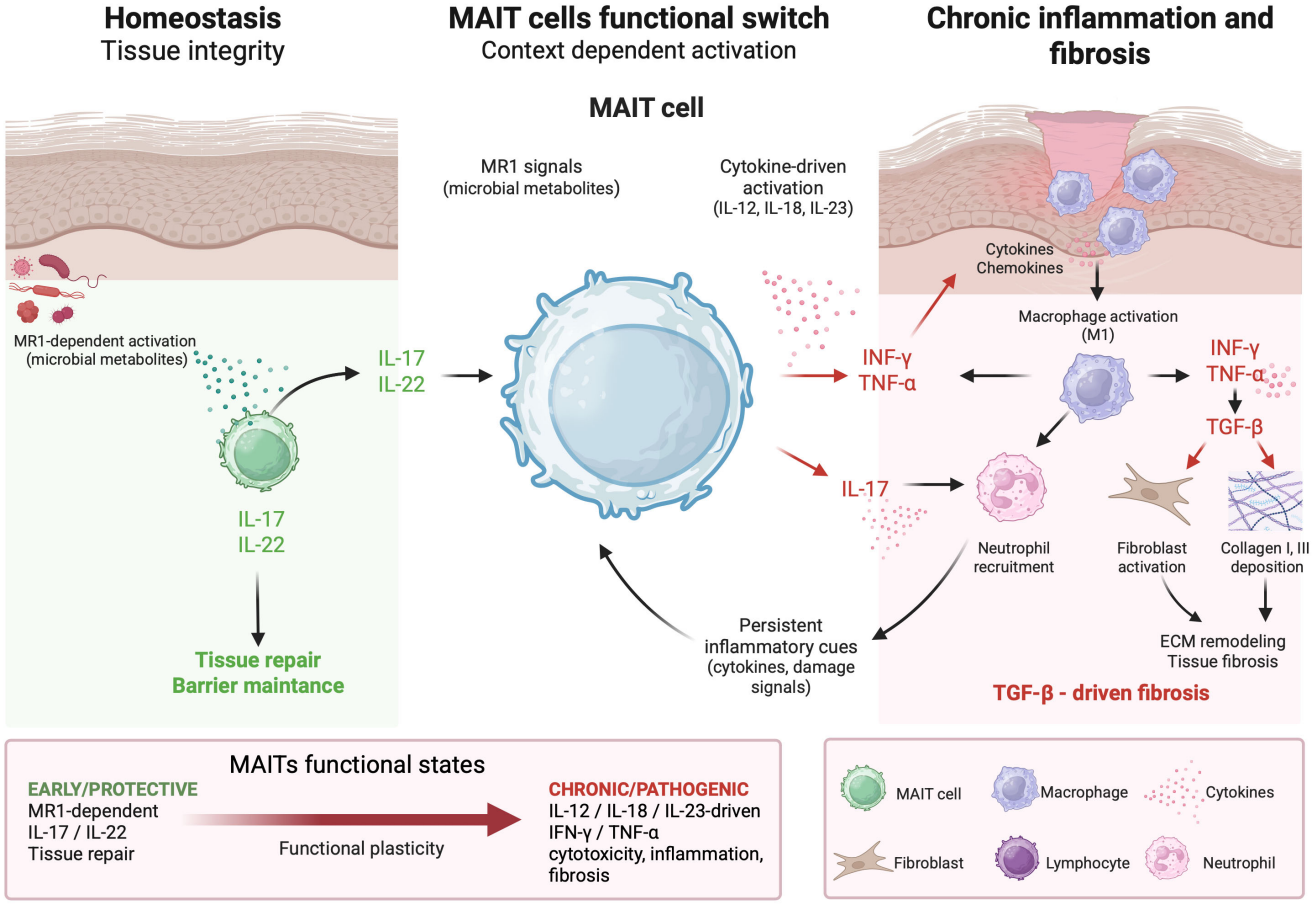
MAIT cells as a key link between inflammation and fibrosis in lichen sclerosus - hypothetical model. This image illustrates the context-dependent functional plasticity of MAIT cells and their potential role in lichen sclerosus (LS). In homeostasis (left panel), MAIT cells contribute to barrier integrity via MR1-dependent activation and production of IL-17 and IL-22; this is established in mucosal immunology but not directly demonstrated in LS. The central panel shows a cytokine-driven functional shift (IL-12, IL-18, IL-23) toward a pro-inflammatory phenotype, which is supported by general MAIT cell biology but remains hypothetical in LS. In the chronic phase (right panel), pathways such as inflammation, macrophage activation, and TGF-β–driven fibrosis are supported by LS data. However, the direct contribution of MAIT cells to these processes is extrapolated from studies in other diseases. Image created by Biorender.com.

A significant component of LS is progressive fibrosis of the skin and mucous membranes. Cytokines produced by MAIT cells—particularly IFN-γ and TNF-α—can modulate fibroblast activity and indirectly promote local TGF-β production by macrophages and epithelial cells (49). TGF-β is a key mediator of fibrogenesis, inducing fibroblast differentiation into myofibroblasts and increasing synthesis of type I and III collagen. Under chronic stimulation, disruption of the balance between extracellular matrix synthesis and degradation leads to persistence of sclerotic lesions (50). Although these pathways are not unique to MAIT cells, their capacity for sustained activation in inflamed tissues suggests that they may contribute to maintaining profibrotic signaling over time. In this framework, MAIT cells are not the sole initiators of fibrosis but participate in a broader cytokine network linking inflammation with tissue remodeling (**Figure 1**). Importantly, the effects of IFN-γ and TNF-α on fibrogenesis are context-dependent, and any contribution of MAIT cells is likely indirect and mediated through chronic inflammation and tissue injury.

Within this framework, a two-phase functional model of MAIT cell involvement in LS can be proposed. In an early, potentially tissue-adaptive phase, MAIT cells may preferentially produce IL-17 and IL-22, supporting epithelial barrier integrity and local immune defense. In contrast, under conditions of persistent inflammation, they may shift toward a phenotype characterized by increased IFN-γ and TNF-α production, enhanced cytotoxicity, and amplification of inflammatory circuits, thereby contributing indirectly to tissue damage and fibrotic remodeling. Importantly, this sequence has not been demonstrated in LS and should be regarded as a testable hypothesis rather than an established mechanism. Its validation would require experimental evidence, including enrichment of MAIT cells within LS lesions (e.g., Vα7.2^+^ MR1-tetramer^+^ populations), upregulation of activation markers such as CD69, HLA-DR, and CD38, altered expression of inhibitory receptors (e.g., PD-1, TIGIT), increased production of IFN-γ, TNF-α, IL-17, IL-22, and cytotoxic mediators such as granzyme B or CD107a upon stimulation, as well as spatial proximity of MAIT cells to fibroblasts, macrophages, or damaged basal keratinocytes. Conversely, the absence of such features or the identification of stable MAIT phenotypes across disease stages would argue against this model.

The role of the inflammatory microenvironment and microbiome cannot be overlooked. Dysbiosis of the skin and mucosal microbiome is increasingly being considered a contributing factor in LS; however, current evidence remains largely associative rather than causal (51, 52). In one study, genetic material was isolated from vulvar skin swabs and sequencing fragments V3–V4 of the 16S rRNA gene, which allowed for the assessment of microbiome diversity and composition and comparison between groups. Patients with vulvar LS showed a lower proportion of *Firmicutes* and a higher proportion of *Proteobacteria*. In healthy women, *Lactobacillus* spp. predominated, while in vulvar LS, *Prevotella* spp. were observed, along with an increased abundance of *Finegoldia*, *Ralstonia*, *Peptoniphilus*, *Anaerococcus*, *Campylobacter*, *Providencia*, *Klebsiella*, *Ezakiella*, and *Escherichia*-*Shigella*, among others (53). These findings indicate compositional shifts but do not establish a direct mechanistic link to immune activation.

MAIT cells are uniquely equipped to detect microbial metabolites via MR1-dependent presentation of riboflavin (vitamin B2) biosynthesis intermediates, such as 5-OP-RU, produced by a broad range of bacteria and fungi (7). However, not all microbial taxa possess the riboflavin biosynthesis pathway required for MR1 ligand production. While some taxa enriched in LS (e.g., *Escherichia coli*, *Klebsiella* spp., and some *Proteobacteria*) are capable of generating such ligands (54, 55), others have not been clearly characterized in this context. Consequently, the functional implications of the observed dysbiosis for MR1-dependent MAIT cell activation in LS remain uncertain.

At present, there is no direct evidence linking microbiome alterations in LS with increased MR1 ligand availability or enhanced MAIT cell activation *in vivo*. Therefore, the relationship between dysbiosis, MR1-dependent signaling, and MAIT cell activation should be considered hypothetical. In parallel, MAIT cells may also be activated in a TCR-independent manner by inflammatory cytokines such as IL-12, IL-18, and IL-23, which are elevated in inflamed tissues and may represent a more immediate mechanism of activation in LS. Taken together, microbiome alterations may modulate MAIT cell activity but cannot currently be regarded as a direct driver of their activation. Further studies integrating microbiome profiling with functional assessment of MR1 ligand production and MAIT cell activity are required.

The role of MAIT cells may also vary across disease stages. In early inflammatory phases, they may contribute to barrier surveillance and tissue repair through IL-17 and IL-22 production. In contrast, during chronic or fibrotic stages, sustained activation may promote a shift toward a proinflammatory and cytotoxic phenotype characterized by increased IFN-γ and TNF-α production, contributing to persistent inflammation and tissue remodeling.

An additional aspect of potential clinical relevance is the association of LS with an increased risk of squamous cell carcinoma. Given the role of MAIT cells in tumor immunity and chronic inflammation, it is conceivable that they may influence the local tumor microenvironment. Depending on the context, MAIT cells could contribute to immune surveillance or, conversely, to chronic inflammatory conditions that facilitate carcinogenesis. At present, this remains entirely speculative and requires further investigation. Future studies examining MAIT cell infiltration and functional phenotype in LS-associated neoplastic lesions may help clarify this potential link.

In summary, MAIT cells may contribute to LS by amplifying Th1 responses, participating in the IL-17 axis, indirectly influencing fibroblast activation via the TGF-β pathway, and responding to cytokine and microbial signals within the altered tissue microenvironment. Their barrier localization, rapid effector function, and unique antigen recognition pathway distinguish them from conventional T cells, supporting a coherent hypothesis linking chronic inflammation with fibrosis, which requires experimental validation.

## 6 Research gaps and future directions

Despite growing interest in the immunological mechanisms of LS, the role of MAIT cells in this disease remains largely unknown. To date, no studies have systematically assessed the abundance and phenotype of this population in patients with LS. It remains unclear whether MAIT cells are increased, decreased, or functionally altered during the course of the disease. Of particular importance is the lack of data regarding the presence of MAIT cells in diseased tissues. In many inflammatory diseases, analysis of the local microenvironment—not just peripheral blood—has allowed us to understand the true role of specific lymphocyte populations. In the case of LS, it is unknown whether MAIT cells accumulate within the lesional skin, whether they participate in the formation of the inflammatory infiltrate, or whether they interact with fibroblasts and antigen-presenting cells. Functional analyses are also lacking. It has not been assessed whether MAIT cells in LS exhibit increased production of proinflammatory cytokines, are activated, or, conversely, are undergoing functional exhaustion. Without such data, it is impossible to determine whether they are a potential driver of the inflammatory process or merely a passive element accompanying the immune response.

However, the most important unknown remains their possible role in the transition from inflammation to fibrosis – a key moment in the pathogenesis of LS. It is unknown whether MAIT cells can influence fibroblast activation, modulate TGF-β secretion, or participate in mechanisms leading to collagen deposition. Understanding this transition is crucial to explaining the chronicity and progression of the disease.

This knowledge gap naturally leads to the need to design studies that will comprehensively assess the presence, phenotype, and function of MAIT cells in LS – both in peripheral blood and in diseased tissues – and determine their potential involvement in the mechanisms linking inflammation and fibrosis.

## 7 Limitations of the MAIT cells hypothesis in LS

The proposed role of MAIT cells in LS is hypothetical and not yet supported by direct experimental evidence. Current assumptions are extrapolated from studies in other autoimmune and fibrosing diseases, which may not fully capture the specific immunological context of LS. Moreover, the functional plasticity of MAIT cells complicates interpretation, as they may exert both pro-inflammatory and tissue-repairing effects depending on the local microenvironment, raising the question of whether they act as true drivers of disease or merely as bystander cells. In addition, the link between MAIT cells and fibrosis remains indirect and has not been demonstrated in LS. Finally, the contribution of other immune populations cannot be excluded. Therefore, the proposed model should be considered a conceptual framework requiring validation in dedicated mechanistic studies.

## 8 Conclusions

LS is a chronic disease characterized by two parallel processes: inflammation and progressive tissue fibrosis and atrophy. Despite advances in understanding the role of classical T lymphocytes and the Th1/Th17 cytokine axis, the precise mechanism responsible for the persistence of chronic inflammation and the transition from the inflammatory phase to predominantly fibrosis remains unclear.

MAIT cells are an interesting population of T lymphocytes because they respond rapidly and robustly, combining features of innate and adaptive immunity. Shortly after activation, they produce large amounts of proinflammatory cytokines such as IFN-γ, TNF-α, and IL-17. These mediators are crucial for both sustaining the inflammatory response and activating cells involved in fibrosis. Therefore, under conditions of chronic inflammation, MAIT cells may not only “amplify” the existing immune response but also influence the tissue microenvironment, promoting the perpetuation of pathological changes.

Importantly, in diseases of a similar nature—combining chronic inflammation with fibrosis—MAIT cells abnormalities have been demonstrated. Both changes in their abundance and functional modifications leading to increased production of proinflammatory cytokines have been described. This suggests that MAIT cells may play a role in diseases in which inflammation and tissue remodeling are closely linked.

Despite these findings, systematic data on the number, phenotype, and activity of MAIT cells are lacking in LS. This represents a significant research gap. This disease is a good model for analyzing the relationship between chronic inflammation and fibrosis, and therefore, the omission of this potentially important cell population may limit a full understanding of its pathogenesis.

## Data Availability

The original contributions presented in the study are included in the article/supplementary material. Further inquiries can be directed to the corresponding author.

## References

[1] De Luca DA Papara C Vorobyev A Staiger H Bieber K Thaci D . Lichen sclerosus: The 2023 update. Front Med (Lausanne). (2023) 10. doi: 10.3389/fmed.2023.1106318 36873861 PMC9978401

[2] Kasprowicz-Furmańczyk M Tadulewicz I Markiewicz A Owczarczyk-Saczonek A . Extragenital lichen sclerosus: A review of the literature. Dermatol Ther. (2026) 16:2233–49. doi: 10.1007/s13555-026-01702-4 41854971 PMC13219593

[3] Kirtschig G Kinberger M Kreuter A Simpson R Günthert A van Hees C . EuroGuiderm guideline on lichen sclerosus—introduction into lichen sclerosus. J Eur Acad Dermatol Venereology. (2024) 38:1850–73. doi: 10.1111/jdv.20082 38822578

[4] Chmielnicki W Sidor A Grykin M Kasprowicz-Furmańczyk M Owczarczyk-Saczonek A . Immunological aspects and quality of life among patients with lichen sclerosus. Skin Health Dis. (2026) 00(00):vzag081. doi: 10.1093/skinhd/vzag081 PMC13425096 42540084

[5] Kirtschig G Kinberger M Kreuter A Simpson R Günthert A van Hees C . EuroGuiderm guideline on lichen sclerosus—treatment of lichen sclerosus. J Eur Acad Dermatol Venereology. (2024) 38:1874–909. doi: 10.1111/jdv.20083 38822598

[6] Bieber AK Steuer AB Melnick LE Wong PW Pomeranz MK . Autoimmune and dermatologic conditions associated with lichen sclerosus. J Am Acad Dermatol. (2021) 85:228–9. doi: 10.1016/j.jaad.2020.08.011 32777320

[7] Nel I Bertrand L Toubal A Lehuen A . MAIT cells, guardians of skin and mucosa? Mucosal Immunol. (2021) 14:803–14. doi: 10.1038/s41385-021-00391-w 33753874 PMC7983967

[8] Cowley SC . MAIT cells and pathogen defense. Cell Mol Life Sci. (2014) 71:4831–40. doi: 10.1007/s00018-014-1708-y 25164578 PMC11113923

[9] Mekinian A Mahevas T Mohty M Jachiet V Rivière S Fain O . Mucosal-associated invariant cells are deficient in systemic sclerosis. Scand J Immunol. (2017) 86:216–20. doi: 10.1111/sji.12585 28727155

[10] DeAngelis LM Cirillo N Perez-Gonzalez A McCullough M . Characterization of mucosal-associated invariant T cells in oral lichen planus. Int J Mol Sci. (2023) 24:1490. doi: 10.3390/ijms24021490 36675003 PMC9860686

[11] Paganelli A Didona D Scala E . Cytokine networks in lichen sclerosus: A roadmap for diagnosis and treatment? Int J Mol Sci. (2025) 26:4315. doi: 10.3390/ijms26094315 40362551 PMC12072692

[12] Kirtschig G . Lichen sclerosus—presentation, diagnosis and management. Dtsch Arztebl Int. (2016) 113(19):337. doi: 10.3238/arztebl.2016.0337 27232363 PMC4904529

[13] Powell J Wojnarowska F Winsey S Marren P Welsh K . Lichen sclerosus premenarche: Autoimmunity and immunogenetics. Br J Dermatol. (2000) 142:481–4. doi: 10.1046/j.1365-2133.2000.03360.x 10735954

[14] Aslanian FM Marques MTQ Matos HJ Pontes LF Porto LCS Azevedo LM . HLA markers in familial lichen sclerosus. HLA-marker bei familiärem lichen sclerosus. (2006) 4:842–7. doi: 10.1111/j.1610-0387.2006.06087.x 17010173

[15] Fergus KB Lee AW Baradaran N Cohen AJ Stohr BA Erickson BA . Pathophysiology, clinical manifestations, and treatment of lichen sclerosus: A systematic review. Urology. (2020) 135:11–9. doi: 10.1016/j.urology.2019.09.034 31605681

[16] Baran W Woźniak Z Batycka-Baran A . IL-17: a novel player in the pathogenesis of vulvar lichen sclerosus. Postepy Dermatol Alergol. (2024) 41:220–5. doi: 10.5114/ADA.2024.139142 38784924 PMC11110226

[17] Gold MC Cerri S Smyk-Pearson S Cansler ME Vogt TM Delepine J . Human mucosal associated invariant T cells detect bacterially infected cells. PloS Biol. (2010) 8:e1000407. doi: 10.1371/journal.pbio.1000407 20613858 PMC2893946

[18] Fergusson JR Smith KE Fleming VM Rajoriya N Newell EW Simmons R . CD161 defines a transcriptional and functional phenotype across distinct human T cell lineages. Cell Rep. (2014) 9:1075–88. doi: 10.1016/j.celrep.2014.09.045 25437561 PMC4250839

[19] Yang JY Wang F Zhou G . Characterization and function of circulating mucosal-associated invariant T cells and γδT cells in oral lichen planus. J Oral Pathol Med. (2022) 51:74–85. doi: 10.1111/jop.13250 34637577

[20] Gherardin NA Souter MNT Koay HF Mangas KM Seemann T Stinear TP . Human blood MAIT cell subsets defined using MR1 tetramers. Immunol Cell Biol. (2018) 96:507–25. doi: 10.1111/imcb.12021 29437263 PMC6446826

[21] Sobkowiak MJ Davanian H Heymann R Gibbs A Emgård J Dias J . Tissue-resident MAIT cell populations in human oral mucosa exhibit an activated profile and produce IL-17. Eur J Immunol. (2019) 49:133–43. doi: 10.1002/eji.201847759 30372518 PMC6519349

[22] Gibbs A Leeansyah E Introini A Paquin-Proulx D Hasselrot K Andersson E . MAIT cells reside in the female genital mucosa and are biased towards IL-17 and IL-22 production in response to bacterial stimulation. Mucosal Immunol. (2017) 10:35–45. doi: 10.1038/mi.2016.30 27049062 PMC5053908

[23] Li J Reantragoon R Kostenko L Corbett AJ Varigos G Carbone FR . The frequency of mucosal-associated invariant T cells is selectively increased in dermatitis herpetiformis. Australas J Dermatol. (2017) 58:200–4. doi: 10.1111/ajd.12456 26940855

[24] Lu B Liu M Wang J Fan H Yang D Zhang L . IL-17 production by tissue-resident MAIT cells is locally induced in children with pneumonia. Mucosal Immunol. (2020) 13:824–35. doi: 10.1038/s41385-020-0273-y 32112047 PMC7434594

[25] Kjer-Nielsen L Patel O Corbett AJ Le Nours J Meehan B Liu L . MR1 presents microbial vitamin B metabolites to MAIT cells. Nature. (2012) 491:717–23. doi: 10.1038/nature11605 23051753

[26] Godfrey DI Koay HF McCluskey J Gherardin NA . The biology and functional importance of MAIT cells. Nat Immunol. (2019) 20:1110–28. doi: 10.1038/s41590-019-0444-8 31406380

[27] Sattler A Dang-Heine C Reinke P Babel N . IL-15 dependent induction of IL-18 secretion as a feedback mechanism controlling human MAIT-cell effector functions. Eur J Immunol. (2015) 45:2286–98. doi: 10.1002/eji.201445313 26046663

[28] Lamichhane R Schneider M de la Harpe SM Harrop TWR Hannaway RF Dearden PK . TCR- or cytokine-activated CD8+ mucosal-associated invariant T cells are rapid polyfunctional effectors that can coordinate immune responses. Cell Rep. (2019) 28:3061–76. doi: 10.1016/j.celrep.2019.08.054 31533031

[29] Kurioka A Ussher JE Cosgrove C Clough C Fergusson JR Smith K . MAIT cells are licensed through granzyme exchange to kill bacterially sensitized targets. Mucosal Immunol. (2015) 8:429–40. doi: 10.1038/mi.2014.81 25269706 PMC4288950

[30] Sundström P Ahlmanner F Akéus P Sundquist M Alsén S Yrlid U . Human mucosa-associated invariant T cells accumulate in colon adenocarcinomas but produce reduced amounts of IFN-γ. J Immunol. (2015) 195:3472–81. doi: 10.4049/jimmunol.1500258 26297765

[31] Samuel CE . Antiviral actions of interferons. Clin Microbiol Rev. (2001) 14:778–809. doi: 10.1128/CMR.14.4.778-809.2001 11585785 PMC89003

[32] Mestan J Digel W Mittnacht S Hillen H Blohm D Möller A . Antiviral effects of recombinant tumour necrosis factor *in vitro*. Nature. (1986) 323:816–9. doi: 10.1038/323816a0 3022155

[33] Wang H Souter MNT de Lima Moreira M Li S Zhou Y Nelson AG . MAIT cell plasticity enables functional adaptation that drives antibacterial immune protection. Sci Immunol. (2024) 9:Eadp9841. doi: 10.1126/sciimmunol.adp9841 39642244

[34] Leng T Akther HD Hackstein CP Powell K King T Friedrich M . TCR and inflammatory signals tune human MAIT cells to exert specific tissue repair and effector functions. Cell Rep. (2019) 28:3077–91. doi: 10.1016/j.celrep.2019.08.050 31533032 PMC6899450

[35] Gold MC Napier RJ Lewinsohn DM . MR1-restricted mucosal associated invariant T (MAIT) cells in the immune response to Mycobacterium tuberculosis. Immunol Rev. (2015) 264:154–66. doi: 10.1111/imr.12271 25703558 PMC4339229

[36] Fan Q Nan H Li Z Li B Zhang F Bi L . New insights into MAIT cells in autoimmune diseases. Biomedicine Pharmacotherapy. (2023) 159:114250. doi: 10.1016/j.biopha.2023.114250 36652733

[37] Böttcher K Rombouts K Saffioti F Roccarina D Rosselli M Hall A . MAIT cells are chronically activated in patients with autoimmune liver disease and promote profibrogenic hepatic stellate cell activation. Hepatology. (2018) 68:172–86. doi: 10.1002/hep.29782 29328499

[38] Paleja B Low A Kumar P Saidin S Lajam A Thanna Bathi LDO . OP0342 altered frequency and function of mait cells in systemic sclerosis revealed by high dimensional mass cytometry and transcriptome analysis. Ann Rheum Dis. (2018) 77:216–7. doi: 10.1136/annrheumdis-2018-eular.6082

[39] Li Y Du J Wei W . Emerging roles of mucosal-associated invariant T cells in rheumatology. Front Immunol. (2022) 13. doi: 10.3389/fimmu.2022.819992 35317168 PMC8934402

[40] Lesturgie-Talarek M Gonzalez V Beaudoin L Frantz C Sénot N Gouda Z . Deficiency and altered phenotype of mucosal-associated invariant T cells in systemic sclerosis. J Scleroderma Relat Disord. (2024) 9:67–78. doi: 10.1177/23971983231209807 38333523 PMC10848929

[41] Wu X Mi Q Chen Y Xu X Xiong X Meng W . MR1‐mediated activation of mucosal‐associated invariant T cells by oral lichen planus‐associated fibroblasts. Oral Dis. (2026). doi: 10.1111/odi.70238 41681071

[42] Wu X Chen S Yang Y Xu X Xiong X Meng W . Circulating mucosal-associated invariant T cell alterations in adults with recent-onset and long-term oral lichen planus. BMC Oral Health. (2024) 24:1183. doi: 10.1186/s12903-024-04959-3 39369184 PMC11453089

[43] Cho YN Kee SJ Kim TJ Jin HM Kim MJ Jung HJ . Mucosal-associated invariant T cell deficiency in systemic lupus erythematosus. J Immunol. (2014) 193:3891–901. doi: 10.4049/jimmunol.1302701 25225673

[44] Hegde P Weiss E Paradis V Wan J Mabire M Sukriti S . Mucosal-associated invariant T cells are a profibrogenic immune cell population in the liver. Nat Commun. (2018) 9:2146. doi: 10.1038/s41467-018-04450-y 29858567 PMC5984626

[45] Mabire M Hegde P Hammoutene A Wan J Caër C Al Sayegh R . MAIT cell inhibition promotes liver fibrosis regression via macrophage phenotype reprogramming. Nat Commun. (2023) 14:1830. doi: 10.1038/s41467-023-37453-5 37005415 PMC10067815

[46] Napier RJ Adams EJ Gold MC Lewinsohn DM . The role of mucosal associated invariant T cells in antimicrobial immunity. Front Immunol. (2015) 6. doi: 10.3389/fimmu.2015.00344 26217338 PMC4492155

[47] Cole S Simpson C Okoye R Griffiths M Baeten D Shaw S . OP0302 mucosal-associated invariant T (MAIT)-cell-derived IL-17A and IL-17F production is IL-23-independent and biased towards IL-17F. Ann Rheum Dis. (2019) 78:232–3. doi: 10.1136/annrheumdis-2019-eular.1914

[48] Xu S Cao X . Interleukin-17 and its expanding biological functions. Cell Mol Immunol. (2010) 7:164–74. doi: 10.1038/cmi.2010.21 20383173 PMC4002915

[49] Mehta H Lett MJ Klenerman P Filipowicz Sinnreich M . Mait cells in liver inflammation and fibrosis. Semin Immunopathol. (2022) 44:429–44. doi: 10.1007/s00281-022-00949-1 35641678 PMC9256577

[50] Biernacka A Dobaczewski M Frangogiannis NG . Tgf-β signaling in fibrosis. Growth Factors. (2011) 29:196–202. doi: 10.3109/08977194.2011.595714 21740331 PMC4408550

[51] Risman SK Milovanovic U Weimer DS Ramadoss T Demory ML . Microbiome dysbiosis in lichen sclerosus: a systematic review. J Clin Gynecol Obstet. (2025) 14:113–21. doi: 10.14740/jcgo1555

[52] Jerkovic Gulin S Almlof O Seifert O Kravvas G Lundin F Bergman Jungestrom M . Genital lichen sclerosus—the role of the vulvar microbiome. Med (Lithuania). (2025) 61:1632. doi: 10.3390/medicina61091632 41011023 PMC12471758

[53] Liu X Zhuo Y Zhou Y Hu J Wen H Xiao C . Analysis of the vulvar skin microbiota in asymptomatic women and patients with vulvar lichen sclerosus based on 16S rRNA sequencing. Front Cell Dev Biol. (2022) 10. doi: 10.3389/fcell.2022.842031 35445011 PMC9014084

[54] Chengalroyen MD Mehaffy C Lucas M Bauer N Raphela ML Oketade N . Modulation of riboflavin biosynthesis and utilization in mycobacteria. Microbiol Spectr. (2024) 12:E03207-23. doi: 10.1128/spectrum.03207-23 38916330 PMC11302143

[55] López-Rodríguez JC Hancock SJ Li K Crotta S Barrington C Suárez-Bonne A . Type I interferons drive MAIT cell functions against bacterial pneumonia. J Exp Med. (2023) 220:E20230037. doi: 10.1084/jem.20230037 37516912 PMC10373297

[56] Litvinova E Bounaix C Hanouna G Da Silva J Noailles L Beaudoin L . MAIT cells altered phenotype and cytotoxicity in lupus patients are linked to renal disease severity and outcome. Front Immunol. (2023) 14. doi: 10.3389/fimmu.2023.1205405 37885889 PMC10598677

[57] Teunissen MBM Yeremenko NG Baeten DLP Chielie S Spuls PI de Rie MA . The IL-17A-producing CD8 + T-cell population in psoriatic lesional skin comprises mucosa-associated invariant t cells and conventional t cells. J Invest Dermatol. (2014) 134:2898–907. doi: 10.1038/jid.2014.261 24945094

